# Two-Dimensional Transition Metal Oxide and Chalcogenide-Based Photocatalysts

**DOI:** 10.1007/s40820-017-0176-y

**Published:** 2017-12-08

**Authors:** Farjana Haque, Torben Daeneke, Kourosh Kalantar-zadeh, Jian Zhen Ou

**Affiliations:** School of Engineering, RMIT University, Melbourne, Australia

**Keywords:** Hydrogen evolution reaction, Pollutant degradation, Water splitting, Layered material, Solar, Carbon reduction

## Abstract

Two-dimensional (2D) transition metal oxide and chalcogenide (TMO&C)-based photocatalysts have recently attracted significant attention for addressing the current worldwide challenges of energy shortage and environmental pollution. The ultrahigh surface area and unconventional physiochemical, electronic and optical properties of 2D TMO&Cs have been demonstrated to facilitate photocatalytic applications. This review provides a concise overview of properties, synthesis methods and applications of 2D TMO&C-based photocatalysts. Particular attention is paid on the emerging strategies to improve the abilities of light harvesting and photoinduced charge separation for enhancing photocatalytic performances, which include elemental doping, surface functionalization as well as heterojunctions with semiconducting and conductive materials. The future opportunities regarding the research pathways of 2D TMO&C-based photocatalysts are also presented. 
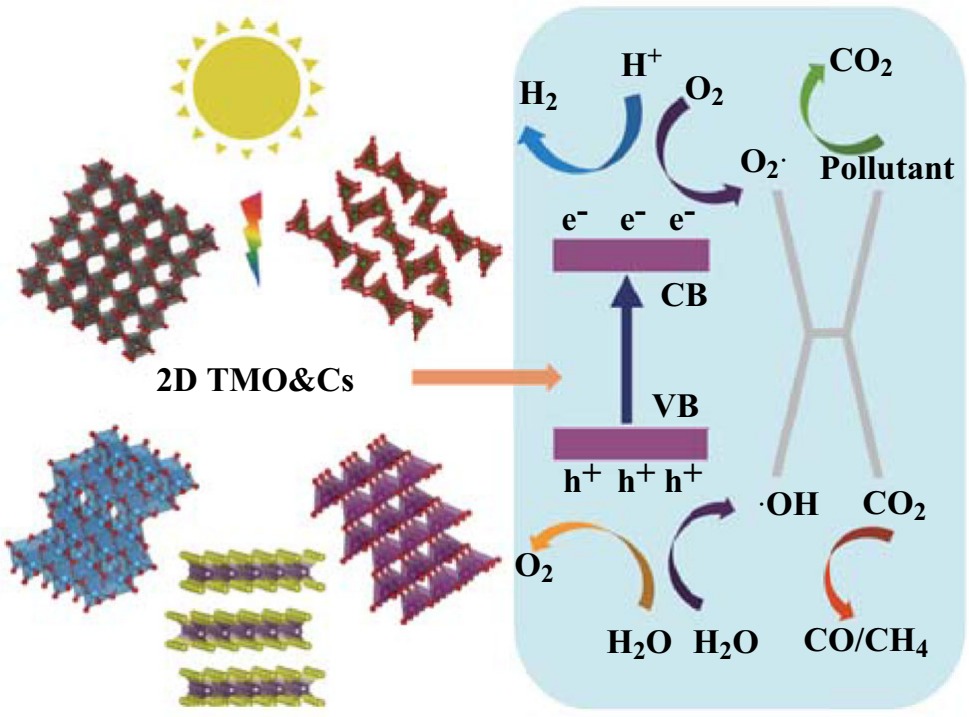

## Highlights

- This review summarizes current two-dimensional (2D) transition metal oxide and chalcogenide (TMO&C)-based photocatalytic systems for hydrogen evolution reactions, organic pollutant degradation, carbon reduction and microbial disinfectants.
- The influences of unique features of 2D TMO&C in terms of crystal and electronic band structures are reviewed regarding their photocatalytic performances.
- The improvement strategies of 2D TMO&C photocatalysts including elemental doping, surface functionalization and heterojunction formation are critically discussed.

## Introduction

Energy shortage and the environmental contamination are the current two of the most common challenges globally, which are mainly attributed to the rapid industrial development and population growth [1]. Therefore, the development of high efficiency, green energy and sustainable technologies for clean energy production and environmental remediation becomes an imperative task [1]. Sunlight is an abundant and easily available natural energy resource, which possesses great potential in driving environment-friendly photochemical reactions. The conversion of solar energy to chemical energy or solar fuels has been considered as one of the most prospective long-term solutions to solve global energy and environmental issues [1, 2]. Photocatalyst is the key to realize such a conversion [3, 4]. Common photocatalytic materials include metal-free organic compounds [5] and semiconductors [6–8], in which most of the interest has been focused on the semiconductor system. In such a system, upon the illumination of an appropriate light source with its energy equal or greater than the bandgap energy of the semiconductor, electrons are excited from the valence band (VB) to the conduction band (CB), inducing the formation of holes in the VB. Then, a fraction of photogenerated electrons and holes migrate to the surface of the semiconductor, while the rest recombine together, releasing the energy in the form of heat or photons [6]. Depending on the chemical potentials of the photoexcitons, various redox reactions can be occurred with absorbed species on the semiconductor surface. For instance, electrons with the potential above that of H_2_/H_2_O can produce H_2_ gas by reacting with the water molecules in the surface of the semiconductor, while holes contribute to the O_2_ production if their potential is below that of O_2_/H_2_O [3]. In the presence of CO_2_ gas together with H_2_O, the photogenerated electrons may participate in reducing CO_2_ to CO, CH_4_ or other forms of carbohydrates, competing with the H_2_ production process due to the fact that the redox potentials for CO_2_ reduction are close to that of H_2_/H_2_O [9]. On the other hand, the holes generated in the VB with the potential below that of OH^−^/H_2_O may react with surface-bound H_2_O or OH^−^ to produce hydroxyl radicals, while the electrons in the CB are picked up by electron accepting species to generate radical anions [10]. The hydroxyl radicals and radical anions are the primary oxidizing species in the photocatalytic oxidation processes, which result in the removal of organic compounds (e.g., dyes, pesticides, phenols and other organic pollutants) and induce oxidative stress to the cell membrane of microbial organism [11–13].

**Fig. 1:**
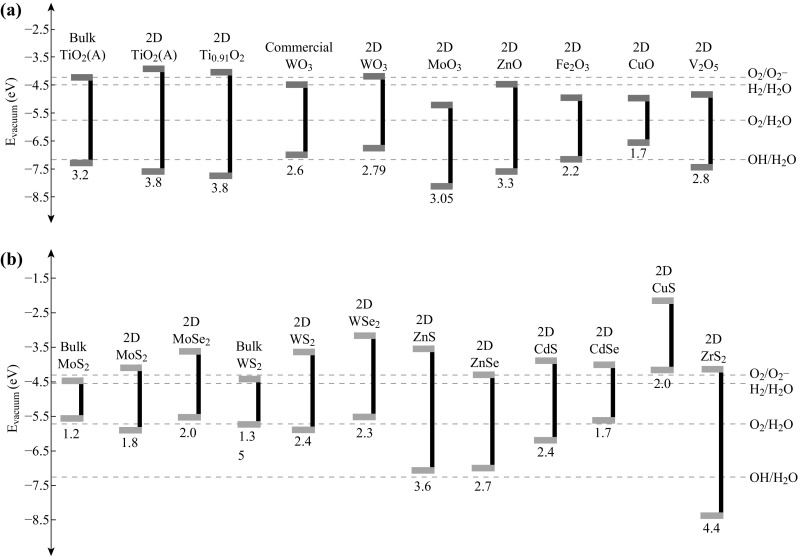
Band structure of currently popular **a** transition metal oxides and **b** transition metal chalcogenides for photocatalytic applications [35, 103, 104, 202–210]

Over the past few decades, a large amount of effort has been devoted to many semiconductors as possible candidates, in which most of them are in the forms of thin film, nanoparticle and one-dimensional (1D) nanostructures [14–16]. Single transition metal oxide (TMO) is the most popular category of photocatalytic semiconductors due to their low cost and excellent chemical stability. The representative candidates are TiO_2_ and ZnO [8, 17–19]. According to Fig. 1, both chemical potentials of the CB and VB of these materials are thermodynamically favorable for many types for photocatalytic applications such as water splitting, pollutant degradation and microbial disinfection. However, their wide optical bandgaps (3.2–3.4 eV) restrict the light absorption only in the ultraviolet region (only 4% of solar spectrum). In addition, they are with low charge carrier mobility and high electron–hole pair recombination rates, which hinder their photocatalytic performances. Therefore, visible light-driven TMOs with relatively narrower bandgap energies are highly desired. For instance, WO_3_, MoO_3_ and V_2_O_5_ have the bandgap energies in the range between 2.6 and 3.0 eV, which lie in the visible light region and hence promote stronger absorption of photons. In addition, they have good electron transport properties that alleviate the characteristic of fast charge recombination which is commonly existed in TMOs [20–22]. But from band structure positions (Fig. 1), those may not be efficient photocatalysts because of their relatively low CB positions, resulting in ineffective consumption of photoinduced electrons during oxygen reduction reactions, and subsequently hinders oxidative degradation of pollutants by holes. Similar challenges are faced by other low bandgap TMOs such as CuO and Fe_2_O_3_ [6, 23–25].

Transition metal chalcogenides (TMCs) are also studied due to their earth abundance. CdS and ZnS are the two most studied materials [26–30]. CdS has a bandgap of 2.4 eV which potentially results in better visible light harvesting compared to many popular TMO-based photocatalysts (Fig. 1). In addition, its CB and VB positions are thermodynamically favorable for many photocatalytic applications. Unfortunately, the separation and transfer of photogenerated electron–hole pairs within the material are inefficient and it suffers from strong photocorrosion effect. In comparison, ZnS is mostly inert in corrosive environment and has the optimum band structures as well. However, its wide bandgap energy (~ 3.6 eV) results in poor visible light absorbability.

In addition to single transition metal oxides and chalcogenides (TMO&Cs), the photocatalytic properties of complex metal oxides and chalcogenides (e.g., binary and ternary metal compounds), nitrides, carbides and phosphides have also been widely studied. However, in this paper, we will not review this category of photocatalytic materials. For more details, readers can refer to some recently published works [31–34].

TMO&Cs in the form of two-dimensional (2D) planar structures are proven to be promising for photocatalytic applications. The larger surface area of 2D planar nanostructures can obviously provide more available active sites for catalytic redox reactions with surface absorbed species [35–37]. In addition, charge migration across both interfaces, i.e., catalyst–electrolyte and catalyst–charge collector, is also promoted, leading to the reduction in interfacial charge transfer resistance and improvement of photocatalytic reaction kinetics [38]. Furthermore, compared to the bulk counterpart, the atomic arrangements at the surface of 2D nanostructures are usually different, possibly due to the surface atomic elongation and structural disordering, which may affect the physical processes of charge transfer at the interface and the defect density [39, 40]. Therefore, the adsorbability of reactant ions or molecular species and the photocorrosion behavior will be significantly altered [39]. More importantly, the 2D planar configuration allows the dominant exposure of one particular facet with distinct atomic arrangement [40, 41], which is more suitable for the separation of photogenerated charge pairs and the utilization of photons. This is due to the fact that the flat band potential and the degree of band bending may be changed at the catalyst–electrolyte interface in the presence of Fermi-level pinning effect [41–43]. It is also well known that the bandgap energies and band positions of certain 2D TMO&Cs are strongly dependent on their thickness and lateral dimensions [44–49]. In many cases, the CB edge will shift toward H_2_ reduction potential or the VB edge will shift toward O_2_ oxidation potential or both happen simultaneously [49]. Therefore, the increased thermodynamic driving force is expected according to the Marcus–Gerischer theory [50, 51]. Moreover, the unique 2D layered structure can be a suitable matrix to induce some special optical phenomena such as plasmonic effect that possibly extends the absorption range of solar spectrum [52, 53].

In this review, we aim to summarize and provide critical discussions on current 2D TMO&C-based photocatalysts compared to their bulk and other dimensional counterparts, with particular focuses on their crystal structures, morphologies and electronic band structures. The methods for synthesizing these photocatalysts are also described in brief. In addition, various approaches on improving the photocatalytic performances of 2D TMO&Cs, such as elemental doping, surface functionalization and heterojunction formation, are critically discussed in terms of their fundamentals and fabrication methods. Finally, a summary of current research progress and perspectives on the challenges and future research directions are given.

In addition, we refer to TMO (e.g., MoO_3_) and TMC (e.g., MoS_2_, MoSe_2_ and MoTe_2_) separately on purpose. This distinction is frequently applied throughout the literature, despite oxygen being a member of the chalcogen group. The origin of this arguably non-intuitive definition is associated with the properties of these inorganic compounds. While a metal’s sulfide, selenide and telluride compounds frequently feature similar properties, the corresponding oxides exhibit stark different properties and stochiometries. As a result, the more similarly behaving compounds are grouped together as chalcogenides, while oxides, being usually the exception to observed trends, are discussed separately.

## The Current Deployment of 2D Single Transition Metal Oxides and Chalcogenides in Photocatalytic Applications

Currently, the deployments of 2D TMO&Cs in photocatalytic applications are relatively limited compared to the nanoparticle and 1D nanostructure systems. 2D TMO&Cs can be classified into two major categories in terms of their crystal structure: (1) layered materials which possess strong lateral chemical bonding in planes but exhibit weak van der Waals interaction between planes and (2) non-layered materials which form atomic bonding in three dimensions and their growth is stopped after a finite numbers of layers with the 2D sheetlike structures [54].

### Layered 2D TMO&Cs

**Fig. 2:**
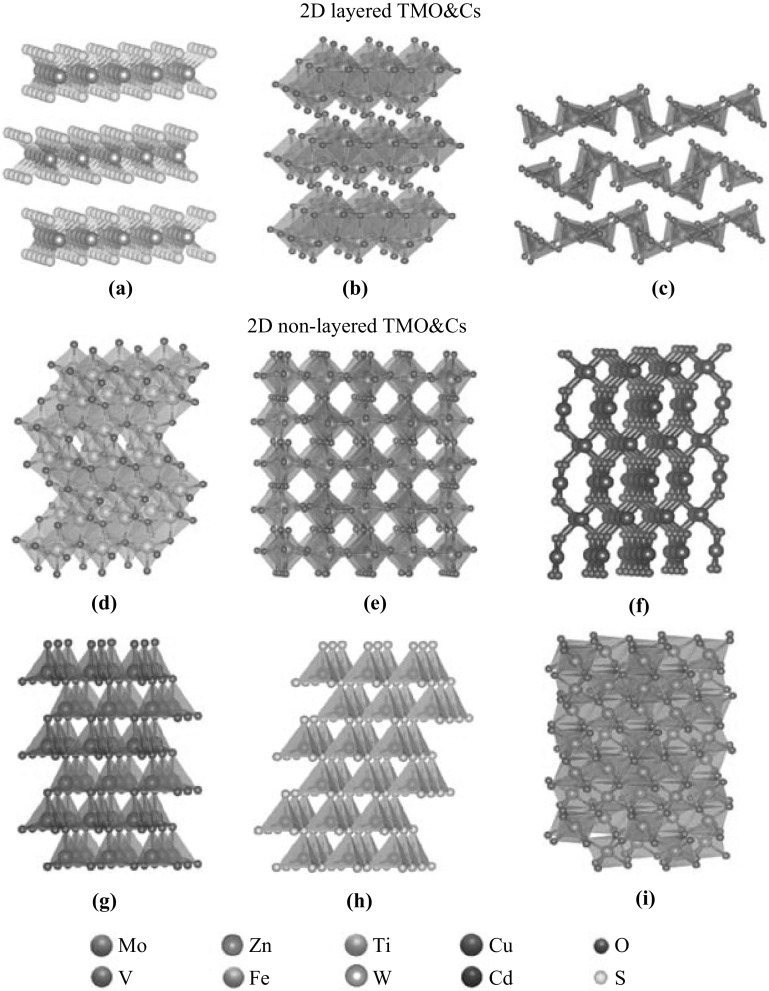
Crystal Structure of **a** hexagonal 2H MoS_2_, **b** orthorhombic MoO_3_, **c** orthorhombic V_2_O_5_, **d** anatase TiO_2_, **e** monoclinic WO_3_, **f** monoclinic CuO, **g** wurtzite ZnO, **h** wurtzite CdS, and **i** rhombohedral Fe_2_O_3_

Mo- and W-based chalcogenides (MX_2_, M = Mo or W and X = S, Se or Te) are typical layered 2D TMCs and emerging photocatalytic materials [55–57]. MX_2_ has a stable hexagonal crystal structure named 2H phase with semiconducting properties. A monolayer of MX_2_ generally consists of two planes of hexagonally arranged X atoms linked to a hexagonal plane of M atoms via covalent bonds (Fig. 2a) [58]. Each M atom is prismatically coordinated by six surrounding X atoms [59]. Such a hexagonal crystal structure can be also seen in a large number of TMCs including the sulfide, selenide and telluride compounds with transition metal element from group IV (Ti, Zr, Hf and so on) and group V (V, Nb or Ta) [37].

Interestingly, the stable 2H semiconducting phase can be transformed to a metastable metallic phase called 1T phase through chemical and electrochemical intercalation of alkaline ions [60]. During the transformation, the planar 2D morphology can be normally retained, but the coordination of M atoms in reference to the surrounding X atoms becomes octahedral [61]. Such a transformation activates the catalytic property of the basal plane in the 2D planar structure, introducing more catalytic sites in the material. Another interesting aspect is that the electronic band structure of MX_2_ is gradually changed from indirect to direct and its bandgap energy enlarges concurrently, when the thickness of MX_2_ is reduced to monolayer [62]. Their CB edges are found to be shifted above the H_2_ reduction potential during the transformation, which implies that its hydrogen evolution reaction (HER) will be more thermodynamically favorable (Fig. 1b). In addition, their bandgap energies lie within the region between 1.6 and 2.4 eV which is ideal for the absorption of visible light. The occurrence of band nesting in their band structures allows highly efficient light absorption at optical gaps other than direct bandgaps, giving rise to absorption as high as 30% at resonance [63]. However, their excited state dynamics studies reveal that the indirect–direct band gap transition in monolayer and few-layer Mo- and W-based TMC results in faster exciton recombination that is dominated by the non-radiative relaxation pathways [64–67], rendering their applications in photocatalysts individually [68]. In addition, the density of surface trap states in monolayer and few-layer structures may affect the trapping of excitons, possibly providing an approach for controlling the exciton dynamics in the 2D systems. Instead, 2D MX_2_ is generally coupled with other visible light-driven photocatalyst as a co-catalyst and electron sink [69, 70]. Detailed discussion will be presented in the later section. The lateral dimensions of 2D MX_2_ used in the photocatalyst are normally within 5 µm, and their thicknesses are less than 10 layers. In comparison, the photocatalytic properties of other layered 2D TMCs are rarely explored. The Zr-, Hf-, Pd- and Pt-based chalcogenides may merit for further investigation due to their narrow bandgaps and suitable band positions [71].

For 2D layered TMO, the research mainly focuses on MoO_3_ and V_2_O_5_. 2D MoO_3_ is generally with the thermodynamically stable orthorhombic phase (α phase). Each individual atomic layer consists of dual-layer planar crystals of distorted MoO_6_ octahedra, held together by weak van der Waals forces in the vertical {010} direction (Fig. 2b) [72]. The internal bonds within the octahedra are dominated by covalent bonds instead. Each of the double layers forms edge sharing zigzag rows along the {001} and corner-sharing rows along the {100} directions, respectively. MoO_3_ also has a metastable phase named β phase but adopts a monoclinic 3D structure, which is not desirable for forming planar crystals [73]. The photocatalytic performance of 2D α-MoO_3_ nanoplatelets with lateral dimension of up to 500 nm and thickness of 20–40 nm is evaluated by Anthony et al. through the degradation of methylene blue (MB) and rhodamine B (RhB) dyes under the irradiation of sunlight. Excellent degradation rates are found to be in the range of 0.8–1.2 mmol (gh)^−1^ [74]. Similarly, 2D V_2_O_5_ generally has an orthorhombic crystal structure, consisting of zigzag double chains of square-based VO_5_ pyramids, bonded together with corner-shared bridge oxygen atoms (Fig. 2c) [75]. Although the photocatalytic H_2_ production rate of 2D V_2_O_5_ nanosheets with lateral dimension of ~ 80 nm is relatively low (~ 0.1 mmol (gh)^−1^) due to its unfavorable band position, however, the enhanced surface area in such a 2D system still demonstrates a significant improvement in reference to the commercial powder counterpart (~ 0.03 mmol (gh)^−1^) [76].

### Non-Layered 2D TMO&Cs

In non-layered 2D TMO&Cs, 2D TiO_2_ undoubtedly is the most studied candidate for photocatalyst followed by great success achieved by its bulk and nanoparticle systems [77]. Generally, TiO_2_ has three most commonly encountered crystalline polymorphs including anatase, brookite and rutile, in which anatase is the most common crystal structure in the 2D configuration [42]. Anatase adopts a tetragonal structure with the share of two opposing edges of each TiO_6_ octahedron to form linear chains along the {001} direction (Fig. 2d) [78]. Interestingly, the mixture of rutile and anatase phases creates a type II band alignment of ~ 0.4 eV, causing the significant decrease in effective bandgap energy and facilitating efficient photoinduced charge separation [79].

It is noted that the particular crystal facet can greatly affect the photocatalytic performance of TiO_2_. Both theoretical and experimental studies found that the facets in the equilibrium state are especially reactive [78]. However, for the conventional anatase TiO_2_ nanocrystal system, it is dominated by either {101} or {001} facets which are less energetically favorable for catalytic reaction [42]. The 2D planar morphology provides the opportunity to engineer the yield of exposed {001} facet in anatase TiO_2_ as large as 95%. In this case, the basal plane of the 2D nanosheet is dominated by the {001} facets on the basis of the symmetries of anatase crystal structure [42]. For the nanosheets with dimension of < 100 nm, it demonstrates a more than four times enhancement in both H_2_ and CH_4_ evolution rates compared to those of conventional anatase nanocrystals [42]. Furthermore, theoretical calculation showed that the {101} and {001} facets exhibited different band edge positions, possibly resulting in forming a surface heterojunction within single TiO_2_ nanosheet and hence facilitating the photogenerated charge separation [43]. When there is Ti deficiency existed in TiO_2_, the corresponding crystal structure may be transformed to an orthorhombic system. Such a group of TiO_2_ derivatives is generally named as “titanate” [77]. Each titanate layer belongs to the lepidocrocite type that consists of two TiO_6_ octahedra connected via edge sharing to form a 2D feature [77]. The bandgap of 2D titanate nanostructure is higher than that of TiO_2_, but the resulted higher CB and lower VB edges are advantageous for photocatalytic performances [77]. Nevertheless, one drawback is the use of more powerful UV light to activate the 2D titanate system. Therefore, it is essential to couple with a visible light-driven photocatalyst for practical applications [77]. Details of the heterostructures will be discussed in the later section.

ZnO is also a core member in the non-layered 2D photocatalyst family. In its bulk form, ZnO crystallizes in either hexagonal wurtzite, cubic zinc-blende structure or both at ambient pressure [80]. Most of the reported 2D ZnO nanostructures are dominated by the wurtzite structure [81]. The structure is composed of two interpenetrating hexagonal close-packed sublattices, each of which consists of one type of atom displaced with respect to each other along the {001} direction (Fig. 2g) [82]. However, scanning tunneling microscopic observations in ultrathin ZnO planar nanostructure reveal that the wurtzite structure may be collapsed and subsequently transformed into a specific 2D arrangement [83]. In addition, the detailed discussion on the specific band structure of 2D ZnO in comparison with the bulk counterpart is rarely found at the moment and further investigation on relevant topics is needed. Both individual 2D nanosheets and 3D hierarchical structures are investigated for their photocatalytic performances toward the degradation of organic dyes [84–86]. The dimension of the structures is generally in the range between 1 and 10 µm and the thickness of individual 2D building block of less than 50 nm. There is generally a one- to two-order enhancement on 2D ZnO regarding the photodegradation performance compared to the nanoparticle system [86].

Similar to ZnO, 2D Zn- and Cd-based chalcogenides also share similar wurtzite crystal structure and have been widely deployed as photocatalysts (Fig. 2h) [87–89]. For example, it is demonstrated that 2D ZnS nanosheets with the lateral dimension of 500 nm exhibit a considerable degradation rate of 0.35 mmol (gh)^−1^ toward methyl orange (MO) under the UV irradiation. It is inactive under visible light exposure due to its large bandgap energy [88]. In comparison, ZnSe has a much narrow bandgap energy of ~ 2.7 eV; hence, it is much popular to be a visible light-driven photocatalyst [90]. 2D ZnSe nanosheets with a lateral dimension of ~ 3 to 5 µm demonstrated a more than 50% enhancement on the RhB degradation rate compared to that of conventional nanoparticle counterpart under visible light illumination [90]. The assembled 3D ZnO hierarchical microspheres also show a MO degradation rate of 40 µmol (gh)^−1^ which is almost 3 times larger than that of nanoparticle system [91]. For the Cd-based 2D chalcogenides, 2D CdS nanosheets with a lateral size of 100–300 nm and a thickness of a few nm demonstrate an excellent H_2_ production rate of ~ 41 mmol (gh)^−1^ under visible light irradiation [92]. Under similar experimental conditions, its 3D flower-shaped derivative with lateral dimension of 300–800 nm also shows an enhanced H_2_ production rate of ~ 9 mmol (gh)^−1^ which is almost 3 times higher than that of nanoparticle system [93]. The H_2_ production rate of flowerlike CdSe ultrathin nanosheet assemblies is more than threefold compared to the commercial powder counterpart. Such an impressive improvement can be ascribed to its enhanced surface area as well as the shift of CB edge above the H_2_/H_2_O potential possibly due to the quantum confinement effect [48].

WO_3_ is another popular non-layered 2D candidate. Monoclinic phase is the most common crystal structure in 2D WO_3_, which contains oxygen corner-sharing WO_6_ octahedrons in a slightly distorted cubic arrangement (Fig. 2e) [94]. Chen et al. propose that the bandgap of the 2D nanosheet (lateral dimension of 1–2 µm and thickness of ~ 9 nm) is slightly larger than the bulk crystal with the positive shift of both CB and VB edges. Therefore, these 2D WO_3_ nanosheets can become an efficient photocatalyst for evaluating CH_4_ at a rate of 1.5 µmol (gh)^−1^ [49]. The 2D WO_3_ nanoplatelets with the lateral dimension of ~ 200 nm also demonstrate a RhB photocatalytic degradation rate of ~ 1 mmol (gh)^−1^ compared to ~ 0.4 mmol (gh)^−1^ of commercial powder [95]. Its 3D flower-shaped assembled structures with average dimension of 1 µm even show a much enhanced photodegradation performance (~ 1.3 mmol (gh)^−1^) [96]. Similar enhancement factor (~ 2.6 mmol (gh)^−1^) is also observed in the slightly hydrated 3D WO_3_ flower-shaped structure [97].

Other non-layered 2D TMO&Cs such as α-Fe_2_O_3_ and CuO are also studied for their photocatalytic properties but to a less extent [98, 99]. α-Fe_2_O_3_ commonly has a rhombohedral structure consisting of FeO_6_ octahedra that share edges with three neighboring octahedra in the same plane and one face with an octahedron in an adjacent plane in the {001} direction (Fig. 2i) [100]. The photocatalytic performance of individual 2D α-Fe_2_O_3_ nanostructure is rarely found in the literature. Instead, the 3D hierarchical nanostructure such as hollow spheres assembled by 2D nanosheets (average dimension of 70 nm) demonstrates the capability of photocatalytic salicylic acid degradation under UV light exposure at a rate of 176 mmol (gh)^−1^, which is more than 40% enhancement than that of nanoparticle [101]. For 2D CuO, the crystal structure belongs to the monoclinic system, in which the Cu atom is coordinated by four coplanar O atoms forming an almost rectangular parallelogram, while the O coordination polyhedron has four Cu atoms at the corners of a distorted tetrahedron (Fig. 2f) [102]. The bandgap of 2D CuO nanosheets (lateral dimension of ~ 1 µm and thickness of < 10 nm) is measured to be ~ 2.0 eV which is much larger than the bulk (~ 1.4 eV) (Fig. 1). Such a bandgap enlargement can be again ascribed to both the quantum size effect and the domination of (002) exposed facet in the 2D configuration [103]. The photodegradation rate of MO under the UV light exposure is 45.5 μmol (gh)^−1^ which is double than that of nanoparticle counterpart [103].

### Synthesis Techniques of 2D TMO&Cs for Photocatalysts

**Fig. 3:**
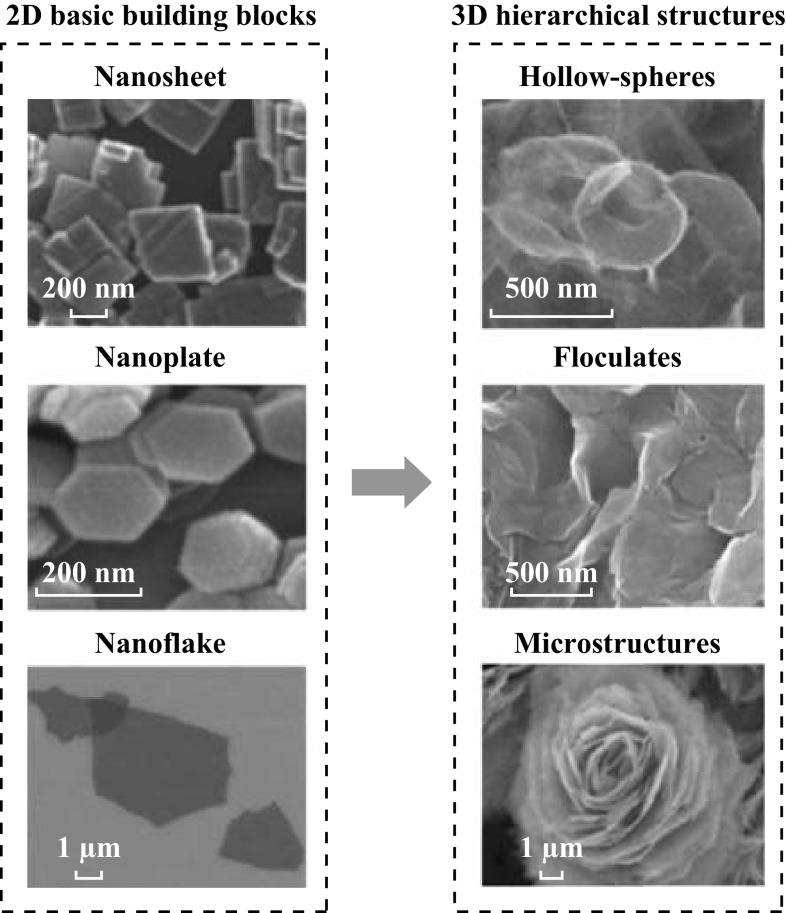
Representative scanning electron microscopic images of the 2D basic building blocks and common 3D hierarchical structures. Reproduced with the permission from Ref. [84, 98, 122, 151, 169, 211]

**Fig. 4:**
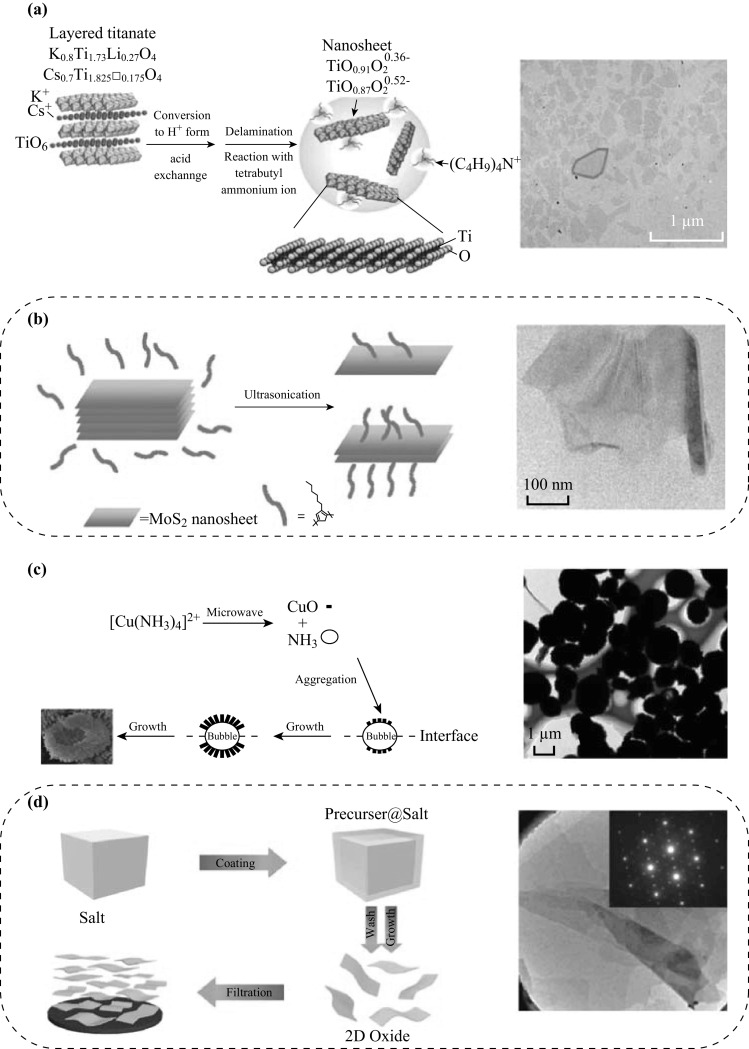
Schematics of synthesis procedures of **a** chemical intercalation, **b** sonication assisted, **c** microwave assisted **d** template assisted for producing 2D TMO&C-based photocatalysts and the corresponding transmission electron microscopic images. Reproduced with permission from Ref. [77, 99, 212, 213]

As the material loading plays an important role in photocatalytic performances, some conventional synthesis approaches for 2D TMO&Cs, such as mechanical exfoliation technique, are not practical to use due to their low production yield (Fig. 3). Chemical vapor deposition (CVD) technique is potentially a suitable candidate for mass production although it is currently not commonly used for preparing 2D photocatalysts. Chemical intercalation (Fig. 4a) and sonication-based (Fig. 4b) liquid-phase exfoliation techniques are generally deployed in 2D layered TMO&C photocatalysts including the most studied MoS_2_ and WS_2_ [104]. Liquid-phase exfoliation technique can also be applied in non-layered 2D TMO&Cs. Taking the example of 2D titanate (e.g., Ti_0.91_O_2_) nanosheets, layered protonated titanate (e.g., H_0.68_Ti_1.83_O_4_·H_2_O) is chosen as the start material due to the non-layered nature of anatase TiO_2_ [105]. The interlayer of the protonated titanate is significantly expanded by replacing the protons with bulky organic ions such as tetra-butylammonium ions (TBA^+^) [105], hence enabling the exfoliation process by applying a weak shear force in the liquid solution [105]. Similarly, 2D WO_3_ nanosheets are obtained from layered tungstate (e.g., WO_3_·2H_2_O) [106].

**Fig. 5:**
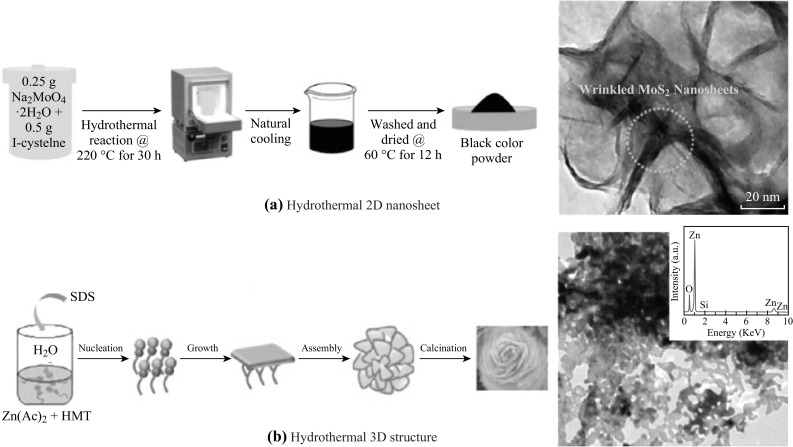
Schematics of hydrothermal synthesis procedures of **a** individual 2D nanostructures and **b** 3D hierarchically assembled structures and their corresponding transmission electron microscopic images. Reproduced with permission from Ref. [84, 214]

The 2D nanostructures can also be obtained hydrothermally/solvothermally using specific precursors for chemical reaction in a pressurized autoclave at elevated temperatures (Fig. 5a) [42, 43, 96, 97]. For example, 2D MoS_2_ nanosheets are synthesized using MoO_3_ and potassium thiocyanate (KSCN) mixture as the source of Mo and S, respectively [107]. For the production of 2D MoO_3_ nanosheets, the precursor consisting of molybdate salt and organic aliphatic acids is used for hydrothermal synthesis [74]. Special attention should be paid to select appropriate reducing agent as unintentional elemental doping may occur in the resulted TMO&Cs.

In addition to the individual 2D nanostructures, functional architectures can be assembled with 2D building blocks using the hydrothermal/solvothermal technique, which are also considered as an important member in 2D TMO&C-based photocatalyst (Fig. 3) [55, 62, 76]. These three-dimensional (3D) assembled structures can significantly improve the photocatalytic performance due to their enhanced active surface area and increased photon–matter interaction via multiple reflection and scattering at the catalyst–electrolyte interface [108]. The selection of capping agent is important for directing the growth of 3D TMO&Cs (Fig. 5b). For example, the selection of thiourea, thioamide or thioacetamide as the chalcogenide during the synthesis results in the 3D MoS_2_ hierarchical structures in the forms of microspheres, ordered flocculates or flowers [62], respectively. The introduction of citric acid as a capping agent facilitates the crystal growth of ZnO along {001} orientation, leading to the formation of 2D nanosheets or 3D hierarchical micro-flowers. However, the absence of citric acid leads to the formation of 1D needlelike morphology due to significant etching along the {001} direction by KOH as a co-reaction agent [109]. The selection of solvent is another important factor to influence the structural and morphological properties of 2D TMO&Cs. Zhang et al. [110] demonstrate that the increase in volume ratio between ethylenediamine and ethylene glycol in the solvent composition transforms the ZnO from the 1D rodlike structures to 3D spherical nano-flowers. Other factors such as temperature and experiment duration are also reported to affect the crystal nucleation behaviors, therefore enabling the precise control of the morphologies and crystallinity of 2D TMO&Cs and their 3D derivative hierarchical structures [111].

It is also reported that high-temperature calcination of TMC can effectively produce 2D TMOs [112]. The application of microwave radiation (Fig. 4c), aqueous-soluble salt templates (Fig. 4d), UV light exposure and electrical field can assist the synthesis of non-layered 2D TMO&C nanostructures including Fe_2_O_3_, WO_3_, CuO and ZnS [49, 88, 99, 101]. Other methods including pyrolysis of metal salts, hot chemical bath and sol–gel have been demonstrated to produce 3D TMO&C hierarchical structures [76].

## Improvement Approach of Photocatalytic Performances in 2D Transition Metal Oxides and Chalcogenides

### Elemental Doping

**Fig. 6:**
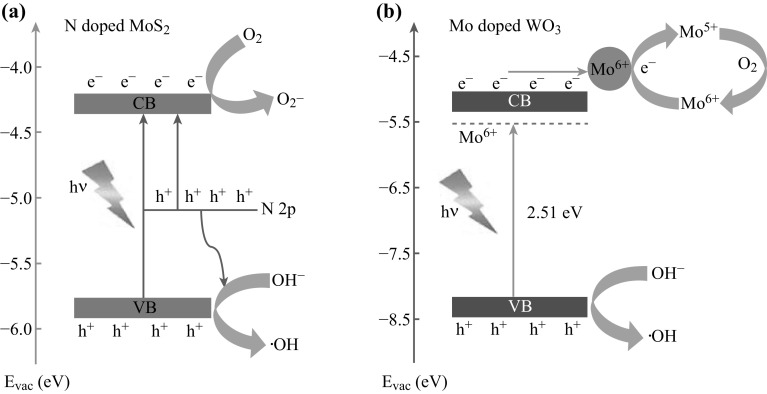
Influence of elemental doping of **a** nonmetal element (N-doped MoS_2_ nanosheets) and **b** metal element (Mo doped WO_3_ nanosheets) toward the band structure. Reproduced with permission from Ref. [117, 122]

Elemental doping is a conventional and effective approach to modify the surface properties and band structures of bulk TMO&Cs for efficient visible light harvesting and enhancement of redox activities [6]. The possible increased mechanical flexibility in some 2D TMO&Cs may result in better incorporation of size-mismatched dopant ions in the crystal compared to the bulk counterpart [113]. The dopants can be generally classified into metal and nonmetal elements [6]. Upon doping with nonmetal elements, the bandgap of TMO&Cs is normally narrowed due to the overlapping between the orbitals of dopant and oxygen/chalcogenide atom [114]. It is also proposed that the formation of localized states within the bandgap by the dopant can contribute to the bandgap narrowing [115]. In this case, the photogenerated electrons can be directly excited from the dopant gap state, instead of the VB edge, to the CB of the material (Fig. 6a). For metal doping, the ionic radius and valence state of the dopant, in reference to those of host metal cation, both play an important role in the determination of occupied sites and electronic properties of the dopant. They may influence the surface properties, position of the Fermi level as well as the conductivity within the matrix [116]. Regardless, the interaction of the dopant with the host lattice leads to the formation of new energy levels either within or beyond the bandgap of TMO&Cs [116]. The light absorption edge can hence be redshifted by electronic transitions from the VB and/or to the CB (Fig. 6). Both metal and nonmetal dopants can also facilitate the separation of photogenerated charge carriers. In particular, when at appropriate concentrations, the dopants can act as deep trap sites for one type of charge carrier, while allowing another one to reach the material surface for desired redox reactions [6].

Nonmetal elements such as N, B, F, P and C have so far been investigated for doping the 2D TMO&Cs. The doping process is mainly realized through the pre-treatment of precursors for synthesis, and the resulted dopant concentration is low which generally causes minimum distortions to the host crystal structure. For example, Liu et al. synthesize the N-doped MoS_2_ hierarchical flower-shaped structure using a sol–gel method, in which thiourea ((NH_2_)_2_CS) is used for the nitrogen dopant source. The bandgap energy of N-doped MoS_2_ (2.08 eV) is slightly lower than those of MoS_2_ nanosheets (2.17 eV), which can be ascribed to the formation of defect state by N dopants in the bandgap of MoS_2_ (Fig. 4a). As a result, the N dopant in MoS_2_ activates its photocatalytic properties, in which the RhB dye is photodegraded in a rate of 134 μmol (gh)^−1^, while no performance is reported in 2D MoS_2_ nanosheets [117].

Similarly, N-doped 2D TiO_2_ nanosheets are produced by a conventional hydrothermal synthesis approach using nitric acid (HNO_3_) as the N dopant source. They have an enhanced visible light absorption in the range from 380 to 500 nm. In addition, the accommodation of N dopants in the TiO_2_ crystal structure favorably enhances the number of exposed {001} facets, which are considered as the highly active catalytic site for TiO_2_ [42, 118]. The resulting H_2_ production rate for the N-doped nanosheets is as high as 17.2 mmol (gh)^−1^, which is about four and two times higher than those of N-doped microcrystals and 2D bare nanosheets, respectively [42, 118]. The selection of tetrafluoroboric acid (HBF_4_) as the hydrothermal reaction agent during the synthesis results in the co-doping of B and F in 2D TiO_2_ nanosheets, which are also dominated by the {001} exposed facets and have a visible light absorption edge appeared at around 516 nm [119].

For 2D ZnO, the introduction of C self-dopants during the hydrothermal synthesis creates a new gap state at the top of the VB and several states mixed with the CB edge, extending the absorption edge to up to 430 nm. The RhB photodegradation rate of the C-doped ZnO is 1.5 μmol (gh)^−1^ that is four times larger than that of commercial powder [120]. However, for P-doped 2D ZnO nanosheets, the bandgap narrowing effect is less obvious. Instead, the dopants play a critical role in obtaining such a 2D planar nanostructure [121].

In comparison, the metal doping is a relatively less common approach for the bandgap engineering of 2D TMO&C structures. Li et al. synthesize the 2D WO_3_ nanosheet doped with Mo ions using ammonium molybdate as the dopant source (Fig. 8a, b). As the ionic radius of Mo is close to that of W, Mo ions are incorporated into the W lattice without disturbing the monoclinic crystal structure. Mo doping created a donor level under the conduction band of WO_3_ to increase the absorption intensity of visible light. The bandgap of Mo-doped 2D WO_3_ decreases from 2.56 to 2.36 eV with the increase in Mo atom (Fig. 6b) content from 0.5 to 10%, The corresponding RhB photodegradation performance is increased from 8 to 12.5 μmol (gh)^−1^ [122]. In comparison, the Mn dopants in 2D CuO nanosheet decrease the host lattice parameters due to the additional strain in the crystal lattice given by different ionic radii between Cu^2+^ and Mn^2+^. 6 wt% of Mn dopant causes a significant reduction in the CuO bandgap energy down to ~ 1.25 eV, resulting in a broad absorption in the region of 200–800 nm. The corresponding photocatalytic MB degradation rate for 2D Mn-doped CuO sheets is almost double in comparison with that of bare CuO powder [123].

In addition to the elemental doping, the incorporation of oxygen vacancies in TMOs also alters the bandgap energy through the formation of localized defect states within the bandgap. The electrical conductivity is improved at the same time, which potentially reduces the charge recombination rate [124]. Furthermore, the partial reduction in the metal oxidation state can act as the trap of electron, hence facilitating the photogenerated charge separation [124]. Certain 2D TMO&Cs can potentially accommodate a large number of oxygen/chalcogen vacancies, significantly increasing the free charge carrier concentration up to ~ 10^21^ cm^−3^ and simultaneously transforming the material from semiconducting to quasi-metallic [125]. Surface plasmon resonance is therefore induced in either the visible or near-infrared spectrum, which greatly improves the solar light harvesting performance of the material [53]. Some pioneer works on standalone 2D MoO_3−x_ nanosheets and core–shell structured 2D MoO_3–x_/MoO_3_ nanosheets have shown improved visible light absorption and demonstrated significantly enhanced photocatalytic performances compared to those of stoichiometric counterpart [126, 127].

### Surface Functionalization

Depending on the specific type of photocatalytic application, functionalization of 2D TMO&Cs with a particular organic group can potentially adsorb more surface species and hence improve the photocatalytic performances. Xue et al. functionalize 2D ZnO nanosheets with amine to enhance the surface capture of CO_2_ molecules for photocatalytic CO_2_ reduction. Monoethanolamine (MEA) is utilized to possess a hydroxyl group for covalent attachment on ZnO and a primary amine group to endow an amine-functionalized surface. The efficient creation of C–N bonding with CO_2_ in the terminal amine groups is believed to activate CO_2_ and produce carbamate. The presence of carbamate can establish direct interactions with Zn^2+^ to receive electrons from ZnO and implement reduction reactions toward CO and CH_4_. From the experiment, both the CO and CH_4_ production rates exhibited by amine-functionalized ZnO nanosheets are double compared to those of bare counterpart [128].

**Fig. 7:**
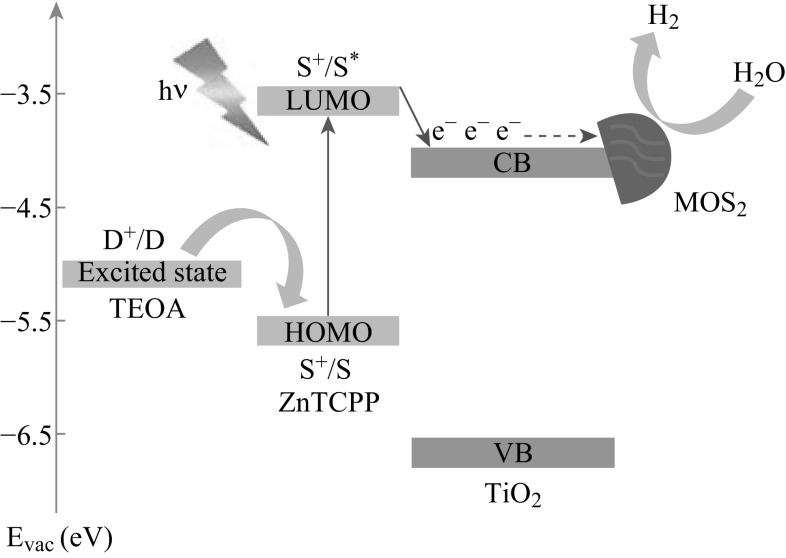
Influence of zinc porphyrin functionalization on the surface of TiO_2_/MoS_2_ in terms of electronic band structure. Reproduced with permission from Ref. [131]

In addition, being aspired by dye-sensitized solar cells, the sensitization of dye molecules on the semiconductor surface is an emerging approach to enhance visible light harvesting [129, 130]. When incorporated into 2D TMO&C systems, the amount of adsorbed dyes can be superior due to the ultra-large surface area offered by the 2D planar geometry [131]. Among various dyes, noble metal-free zinc porphyrin complexes have been studied intensively as photosensitizers for dye-sensitized solar cells with their highest conversion efficiency of > 10% [132]. Due to the delocalized *p* electrons, zinc porphyrin has strong absorption in visible light region, outstanding photochemical stability and more importantly, suitable redox potentials for electron injection and dye regeneration, making it attractive as photosensitizers [133]. In a few recent reports, zinc porphyrin has been incorporated into the 2D MoS_2_ nanosheet heterostructured with TiO_2_ and ZnO [131, 133]. In this system, the electrons are excited from the highest occupied molecular orbital (HOMO) to the lowest unoccupied molecular orbital (LUMO) of zinc porphyrin complexes to form an excited intermediate upon visible light illumination (Fig. 7). Due to the relatively negative oxidation potential of the dye, the generated electrons are injected from the excited zinc porphyrin complexes to TMO. Then, 2D MoS_2_ nanosheet is utilized as a co-catalyst, which collects the excited electrons from the TMO for HER. The enhancement factor of photocatalytic H_2_ production when incorporated porphyrin complexes can exceed one order compared to the bare MoS_2_/TMO heterostructure [131, 133]. In addition to zinc porphyrin complexes, eosin Y is also utilized in 2D MoS_2_ nanosheet–graphene composites [134]. Although superior photocatalytic hydrogen evolution is demonstrated, the longevity of this dye-functionalized photocatalytic system may be questioned due to the short lifetime of the dye.

### Heterojunctions

#### Heterojunction with Semiconductors

**Fig. 8:**
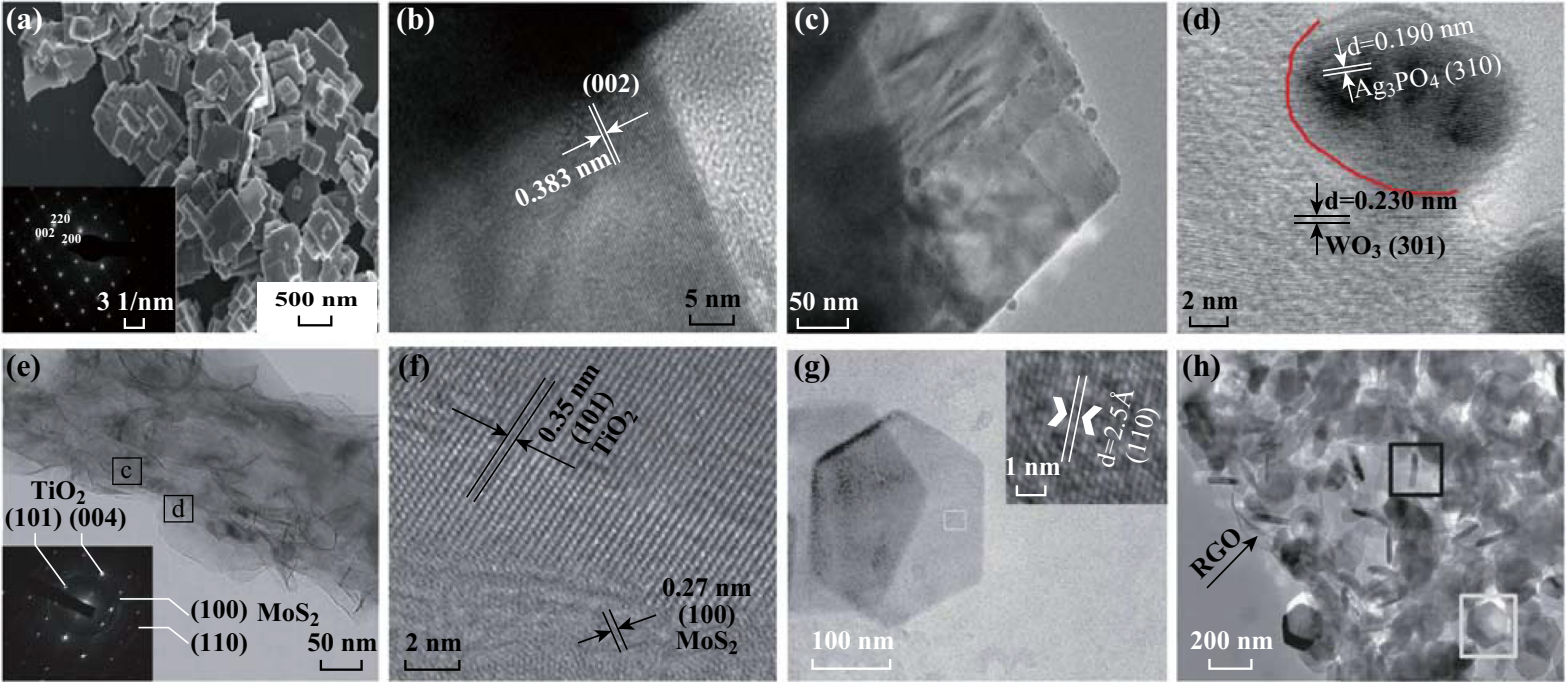
Representative low- and high-resolution transmission electron microscopic images of **a, b** 2D-doped TMO&C nanosheets, **c, d** 2D–0D heterojunctions, **e, f** 2D–1D heterojunctions and **g, h** 2D–2D heterojunctions. Reproduced with permission from Ref. [56, 98, 122, 168]

**Fig. 9:**
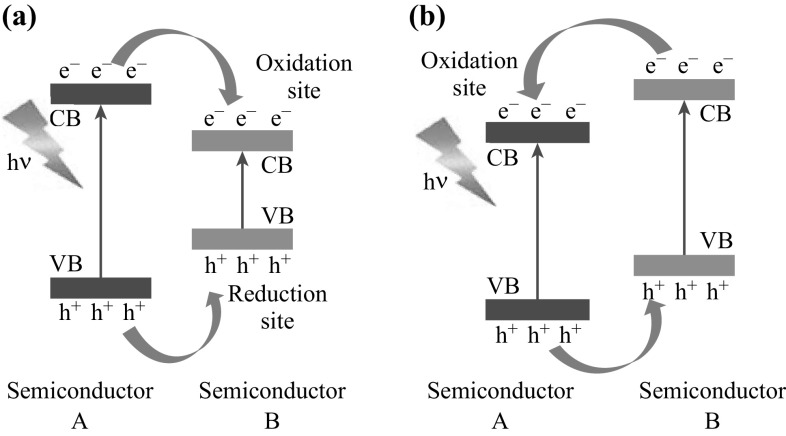
Semiconducting heterojunctions with **a** type I and **b** type II band alignments. Reproduced with permission from Ref. [215]

In addition to the limited visible light harvesting performance, fast recombination of photogenerated charge carrier in many individual 2D TMO&Cs is a great concern. Constructing a 2D TMO&C-based heterojunction with a properly selected semiconductor can address such a concern [6]. A good matching of CB and VB energetic levels between 2D TMO&Cs and the semiconductor can produce an effective transfer pathway for photogenerated charge carriers from one to the other. The most popular approach is the type II band alignment (Fig. 9b). The photogenerated electrons transfer from the more positive CB edge to the less positive one, while the holes transfer from the more negative VB edge to the less negative one, hence realizing the spatial charge carrier separation [135]. However, many other factors, such as defect density and crystallinity, can significantly alter the band structures of the materials hence influencing the coupling efficiency [6]. Furthermore, the dimensionality and size difference between coupled semiconductors may also be important for hetero-interfacial contacts [108]. Compared to other 2D/low-dimensional counterparts, the 2D/2D heterostructure (Fig. 8g, h) exhibits better stability and coupling heterointerfaces due to the large contact surface and short exciton diffusion length in the contact, which facilitates the transfer and separation of photoexciton pairs [108].

However, the investigation on 2D/2D semiconducting heterojunctions with type II band alignment is still relatively limited. Most attention has so far been paid to 2D MoS_2_-based heterojunctions due to its high electron mobility and excellent electrocatalytic HER performance. Graphic carbon nitride (g-C_3_N_4_) is a popular 2D semiconductor that forms heterojunction with 2D MoS_2_ due to its optimum bandgap energy (2.7 eV) for visible light harvesting as well as proper CB and VB positions for efficient water splitting [136]. In comparison with 2D MoS_2_, the CB edge potential of g-C_3_N_4_ is − 2.8 V (vs. vacuum) which is less negative than that of MoS_2_ (− 4.2 V), allowing the migration of electrons from g-C_3_N_4_ to MoS_2_. On the other hand, the hole generated from MoS_2_ can be transferred to g-C_3_N_4_ due to more negative VB potential of MoS_2_ (− 6 V) compared to g-C_3_N_4_ (− 5.5 V), hence achieving efficient charge separation. Therefore, the drawbacks of individual g-C_3_N_4_-based photocatalyst, such as limited delocalized conductivity and high charge recombination rate, can be significantly alleviated when forming the heterojunction with 2D MoS_2_ [55]. In addition, due to the broad visible-NIR absorption range of 2D MoS_2_, the heterojunction may have enhanced absorbability of solar light spectrum. The corresponding H_2_ evolution rate of g-C_3_N_4_ loaded with 1 wt% 2D MoS_2_ nanosheets is 35.6 µmol (gh)^−1^ which is one order higher than that of g-C_3_N_4_ [136]. There are also a three times and 20% enhancement on photocatalytic RhB and MO degradation for 2D g-C_3_N_4_/MoS_2_ compared to g-C_3_N_4_, respectively [55, 137].

2D ZnO nanosheet is another candidate to form 2D/2D semiconducting heterojunction with 2D MoS_2_ [138]. While their coupled band structure is identified as type II band alignment by some researchers, it should be noted that the potentials between their CB edges are very close (− 4.4 V for ZnO vs. − 4.2 for MoS_2_) [121, 138]. This implies that attention should be paid during the synthesis of 2D ZnO/MoS_2_ as minor modifications of the surface may alter the CB edges and hence lead to the transformation to type I band alignment, which is less favorable for charge separation (Fig. 9a). Furthermore, the CB edge potential of 2D MoS_2_ strongly depends on its thickness [139]. Therefore, the influence of 2D MoS_2_ thickness in the heterojunction on photocatalytic performances may merit for further investigation. In addition to single TMO&Cs for constructing 2D/2D heterojunctions, binary 2D TMO&C compounds, including CuInS_4_ and ZnInS_4_, have also been investigated owing to their relatively narrow bandgap (1.5–1.9 eV for CuInS_4_ and 2.4–2.5 eV for ZnInS_4_) and suitable band structures when coupling with 2D MoS_2_ [140, 141]. It is found that a loading of 2 wt% 2D MoS_2_ in CuInS_2_-based heterojunction leads to a H_2_ evolution rate of 316 µmol (gh)^−1^, which is two order higher than CuInS_2_ and three times larger than Pt-loaded CuInS_2_ [141]. Similarly, a double enhancement on the H_2_ production rate is observed in the 3 wt% 2D MoS_2_/ZnInS_4_ heterojunction in reference to Pt-loaded ZnInS_4_ [140].

The heterojunctions of 2D MoS_2_ with other low-dimensional TMO&Cs have also been widely investigated. Popular candidates include TiO_2_ [56, 69, 70, 142, 143], CdS [144, 145], ZnS [146], MoO_3_ [112], ZnO [147] and CuS [148]. The detailed photocatalytic performances are shown in Tables 1, 2, 3, 4, 5 and 6. In these systems, 2D MoS_2_ nanosheet is considered as an electron sink candidate similar to the role of graphene, for achieving efficient charge separation in the heterojunction [148]. However, in the view of their band structures, type I band alignment instead of type II is generally achieved in these heterojunctions. The VB edge potential of MoS_2_ is less negative than those of aforementioned coupled materials although its CB edge potential is more negative (Fig. 9). Such a band mismatch is less favorable for charge separation as the photogenerated electron–hole pairs from the coupled materials are both transferred to MoS_2_. The replacement with metallic 1T MoS_2_ may be a viable solution, and its detailed mechanism will be discussed in the later section.

**Table 1:** Summary of photocatalytic H_2_ evolution performance of 2D bare TMO&Cs

Material	Dimension/thickness	Light source	Reaction solution	H_2_ PR [% mmol (gh)^−1^]	Comparison	Ref.
TiO_2_ NS	70 nm/2 nm	Xe lamp	Methanol	6	1.3 (TiO_2_ Cuboids)	[42]
V_2_O_5_ NS	–	Xe lamp	Methanol	0.022	–	[76]
CdS NS	100–300 nm/4 nm	Visible light	Na_2_S/Na_2_SO_3_	41.1	–	[92]
CdS flower	5 µm/10–100 nm	Xe lamp	Lactic acid and water	9.3	2.6 (CdS NS)	[93]
CdSe flower	5 µm/4.8 nm	Visible light	Na_2_S/Na_2_SO_3_	56.4	0.075 (CdS NR); 44 (CdS QD)	[48]

*PR* production rate, *NS* nanosheet, *NR* nanorod, *QD* quantum dot

**Table 2:** Summary of photocatalytic H_2_ evolution performance of 2D modified TMO&Cs

Material	Dimension/thickness	Light source	Loading	Reaction solution	H_2_ PR (% mmol (gh)^−1^)	Comparison	Ref.
*Elemental doping*							
N-TiO_2_ NS	80 nm/20 nm	UV–Vis light	NA	Ethanol	0.865	0.211 (N-TiO_2_ MC)	[118]
*Heterojunction with semiconductors*							
TiO_2_ NS/MoS_2_ NS	100 nm/12 nm	Xe lamp	0.5 wt% MoS_2_	Methanol	2.145	0.061 (TiO_2_)	[193]
MoS_2_ NS/CdS NP	–	Xe lamp	0.2 wt% MoS_2_	Lactic acid	5.3	–	[194]
1T-MoS_2_ NS/TiO_2_ NC	–	UV light	–	Methanol	2	0.6 (2H-MoS_2_)	[188]
MoS_2_ NS/ZnO NP	30–50 nm/–	Xe lamp	1 wt% MoS_2_	Na_2_S/Na_2_SO_3_	0.765	0.052 (ZnO)	[147]
MoS_2_ NS/CuInS_2_ NP	4 µm/80 nm	Xe lamp	3 wt% MoS_2_	Na_2_S/Na_2_SO_3_	0.316	0.011 (CuInS_2_)	[141]
MoS_2_ NS/TiO_2_ NF	–	Xe lamp	60 wt% MoS_2_	Na_2_S/Na_2_SO_3_	1.6	–	[142]
MoS_2_/TiO_2_ NW	–	Visible light	–	TEOA-H_2_O	16.7	–	[70]
1T-WS_2_/TiO_2_ NP	100 nm/–	Xe lamp	–	Distilled water and Methanol	2.57	0.225 (2H-WS_2_/0D TiO_2_)	[55]
ZnS/CuS NP	20 nm/–	Visible light	–	Na_2_S/Na_2_SO_3_	4.147	–	[164]
TiO_2_ NS/CdS QD	–/0.7 nm	Visible light	–	Na_2_S/Na_2_SO_3_	0.1	–	[153]
MoS_2_/N-rGO NS	80 nm/–	Visible light	–	Ethanol	0.025	–	[162]
*Heterojunction with conductive materials*							
TiO_2_ NS/Au–Pd NP	200 nm/–	UV–Vis light	0.3 wt% TIO_2_	Methanol	0.526	–	[177]
ZnO NS/Au/CdS NP	5 µm/100 nm	W lamp	–	Na_2_S/Na_2_SO_3_	0.608	–	[165]
MoS_2_ NS/Ag NP	–	Xe lamp	20 wt% MoS_2_	Na_2_S/Na_2_SO_3_	36	24 (MoS_2_)	[175]
MoS_2_-Graphene NS/TiO_2_ NC	7–10 nm/–	UV light	–	Ethanol/water	2.06	0.0625 (TiO_2_ NC)	[69]
MoS_2_-graphene NS/ZnS NP	–	Xe lamp	2 at % MoS_2_	Na_2_S/Na_2_SO_3_	2.26	0.12 (ZnS)	[146]
MoS_2_ NS/g-C_3_N_4_ NS	–	Xe lamp	0.5 wt% MoS_2_	Methanol	17.8	–	[136]
WS_2_ NS/g-CN NS	–	Xe lamp	0.3 at % WS_2_	Lactic acid	0.12	–	[150]
ZnS NS/Ag_2_S NP	–/20 nm	Xe lamp	–	Na_2_S/Na_2_SO_3_	0.105	–	[192]
*Surface functionalization*							
MoS_2_ NS/ZnTCPP/ZnO NS	–	Visible light	0.5 wt% MoS_2_	Triethanolamine	0.750	–	[133]
MoS_2_ NS/ZnTCPP/TiO_2_ NP	–	Xe lamp	1 wt% MoS_2_	TEOA	0.102	–	[131]

*PR* production rate, *NS* nanosheet, *NP* nanoparticle, *NF* nanofiber, *NW* nanowire, *QD* quantum dot, *MC* microcrystal, *NC* nanocrystal

**Table 3:** Summary of carbon reduction performances of 2D TMO&Cs and their composites

Material	Dimension/thickness	Light source	Reaction solution	Hydrocarbon PR (µmol (gh)^−1^)	Ref.
*Single2D TMO&Cs*					
WO_3_ NS	9 nm/4–5 nm	Xe lamp	Distilled water	1.5 (CH_4_)	[49]
TiO_2_ NS	80 nm/30 nm	–	Deionised water	1.35 (CH_4_)	[43]
TiO_2_ NS	70 nm/2 nm	UV light	Deionised water	5.8 (CH_4_)	[42]
*Heterojunction with semiconductors*					
Ti_0.91_O_2_ NS/CdS NP	–/0.75 nm	Visible light	Deionised water	10 (CH_4_)	[169]
*Heterojunction with conductive materials*					
TiO_2_ NS/r-GO NS	0.1–1 μm/0.75 nm	Visible light	Deionised water	1.14 (CH_4_); 8.91 (CO)	[187]
*Surface functionalization*					
Amine–ZnO flower	–	UV light	Ultrapure water	1.1 (CH_4_); 6.33 (CO)	[128]

*PR* production rate, *NS* nanosheet, *NP* nanoparticle

**Table 4:** Summary of pollutant degradation performances of 2D bare TMO&Cs

Material	Dimension/thickness	Light source	Reaction solution	DR (% μmol (gh)^−1^)	Comparison	Ref.
WO_3_ flower	3–5 μm**/**25 nm	Xe lamp	RhB	1.3	–	[96]
WO_3−*x*_/WO_3_·H_2_O NW	–	Visible light	MB	70.3	–	[124]
WO_3_·0.33H_2_O microsphere	4 μm**/**200 nm	UV–Vis–NIR light	RhB	2.6	–	[97]
TiO_2_ NS	130 nm**/**8 nm	–	MO	16.71	–	[195]
ZnO flower	1–2 µm/–	Hg lamp	RhB	125	–	[84]
ZnO hollowsphere	–/160 nm	Hg lamp	RhB	10.5	–	[196]
Fe_2_O_3_ hollowsphere	70 nm/–	UV light	MO	458	–	[101]
MoO_3−*x*_ nanoplates	50–70 nm/> 20 nm	Sunlight	MB	1250	–	[74]
CuO NS	100–1000 nm/10–30 nm	UV light	MO	45.5	19.06 (NP)	[103]
CuO hollowsphere	500 nm/–	UV–Vis light	RhB	4.5	–	[99]
ZnS NS	–	Hg lamp	MO	500	–	[88]
ZnSe microsphere	1 μm/80 nm	Xe lamp	MO	40.41	16.66 (NP)	[91]
WS_2_ NS	2–4 μm/100 nm	UV–Vis–NIR light	MO	7.5	–	[158]
CuS microsphere	2 μm/1–10 nm	Sunlight	MB	218.07	143.03 (NP)	[197]
ZrS_2_ NS	10–30 nm/7 nm	Xe lamp	4-NP	31.68	–	[198]

*DR* degradation rate, *NS* nanosheet, *NP* nanoparticle, *NW* nanowhisker

**Table 5:** Summary of pollutant degradation performances of 2D modified TMO&Cs

Material	Dimension/thickness	Light source	Loading	Reaction solution	DR (% μmol (gh)^−1^)	Comparison	Ref.
*Elemental doping*							
Mo-WO_3_ NS	400–1600 nm/150 nm	Visible light	NA	RhB	12.5	–	[122]
B, F-codoped TiO_2_NS	10 nm/2.5 nm	Xe lamp	NA	MB	18	–	[119]
C-ZnO flower	9 µm/~ 10 nm	Xe lamp	NA	RhB	1.5	–	[120]
ZnO NS/P-MoS_2_ NS	–	Sunlight	NA	MB	222	–	[121]
Mn-CuO NS	–	Xe lamp	NA	MB	20.26	11.25 (CuO)	[123]
N-MoS_2_ flower	–	Visible light	NA	RhB	134.2	–	[117]
*Heterojunction with semiconductors*							
WO_3_ NS/Ag_3_PO_4_ NP	–	Xe lamp	–	MB	85.5	–	[168]
Ti0_.87_O_2_ NS/CdS pillar	100–500 µm/–	Xe lamp	–	MB	58	0.078 (N-TiO_2_)	[151]
ZnO NS/WO_3_ NR	–	Xe lamp	–	MB	533	–	[154]
MoS_2_ NS/CdS NP	–	Xe lamp	2 mol % MoS_2_	MB/RhB	40	–	[144]
MoS_2_ flower/CdS NP	800 nm/–	Xe lamp	–	MB	97	–	[98]
MoS_2_NS/CuS NP	–	Visible light	–	MB	104.21	–	[148]
Fe_3_O_4_@MoS_2_ Core − Shell	–	Blue light	–	4-NP	285	–	[199]
MoS_2_ NS/Ag_3_PO_4_ NP	–	Xe lamp	1 wt% MoS_2_	RhB	93.9	–	[68]
MoS_2_ NS/Ag_3_PO_4_ NP	–	Xe lamp	0.65 wt% MoS_2_	RhB	18.78	–	[166]
MoS_2_-r-GO NS/Ag_3_PO_4_ NP	–	Xe lamp	0.02 wt% MoS_2_-r-GO	Phenol	1593.8	–	[167]
MoS_2_ NS/TiO_2_ NF	120–300 nm/–	UV light	–	MO	497.53	26.18 (MoS_2_);	[143]
MoS_2_ NS/TiO_2_ NR	–	Xe lamp	50 wt% MoS_2_	RhB	939.4	817.3 (MoS_2_); 328.8 (TiO_2_)	[56]
ZnS NS/ZnO NP	400–1000 nm/40 nm	W lamp	–	MB	6.4	4.6 (ZnS)	[155]
WS_2_/WO_3_ NP	–	Visible light	–	MB	378.9	–	[157]
*Heterojunction with conductive materials*							
TiO_2_ NS/carbon QD	45–55 nm/6 nm	Visible light	–	RhB	19.83	8.3 (TiO_2_ NP)	[159]
TiO_2_ NS/g-C_3_N_4_ NS	38 nm/6 nm	UV–Vis light	–	MB	4.5	–	[149]
ZnO flower/Ag NP	–/10–19 nm	Hg lamp; Xe lamp	–	RhB	3.2	0.88 (ZnO NP)	[174]
ZnO NS/Ag NP	–	UV light	–	MO	389.5	–	[176]
Fe_2_O_3_ NS/r-GO NS	200 nm/–	Xe lamp	–	RhB	1.4	–	[98]
MoS_2_ NS/g-C_3_N_4_ NS	–	–	0.05 wt% MoS_2_	MO	40.7	–	[55]
MoSe_2_ NS perpendicular to r-GO NS	–	Xe lamp	–	MB	70.34	15.4 (MoSe_2_ NS/r-GO NS)	[181]
CoS NS/2D r-GO NS	~ 200–300 nm/~ 10–20 nm	Visible light	26.2 wt% CoS	MB	47.41	28.13 (CoS)	[182]

*DR* degradation rate, *NS* nanosheet, *NP* nanoparticle, *NR* nanorod, *NF* nanofiber, *QD* quantum dot

**Table 6:** Summary of antimicrobial disinfection performances of 2D TMO&Cs and their compounds

Material	Dimension/thickness	Light source	Target	Destruction rate	Ref.
*Elemental doping*					
B, F-codoped TiO_2_ NS	10 nm/2.5 nm	Xe lamp	*E. coli*	99.5%	[119]
*Heterojunction with semiconductors*					
MoS_2_ NS/ZnO flower	–	UV–Vis light	*E. coli*	45%	[138]
ZnO NS/NaYF4:Yb, Tm NC	–	Xe lamp	*E. coli*, *C. albicans*, *S. aureus*	MIC-50 µg mL^−1^, 50 µg mL^−1^, 100 µg mL^−1^	[200]
*Heterojunction with conductive materials*					
TiO_2_ NS/Ag NP	–	UV light	*Pseudomonas* sp. and *Bacillus* sp.	0.07% and 8 × 10^−4^ %	[201]

*NS* nanosheet, *NP* nanoparticle, *NC* nanocrystal, *MIC* minimal inhibitory concentration

For other 2D TMO&Cs instead of MoS_2_, there are also occasional reports on 2D/2D semiconducting heterojunctions with type II band alignment, including 2D g-C_3_N_4_ coupled with 2D TiO_2_ and WS_2_ nanosheets [149, 150]. However, the formation of 2D/0D (Fig. 8c, d) and 2D/1D (Fig. 8e, f) semiconducting heterostructures is much more popular. Representative examples are 2D Ti_0.91_O_2_ nanosheet/0D CdS quantum dot [151–153], 2D ZnO nanosheet/1D WO_3_ nanorod [154], 2D ZnS nanosheet/0D ZnO nanograin [155], 2D ZnSe nanosheet/1D ZnO nanorod [156], 2D WS_2_ nanosheet/0D WO_3_ nanoparticles [157] and 2D WS_2_ nanosheet/0D TiO_2_ nanoparticles [158]. Heterojunctions formed with 0D up-converted materials (e.g., carbon dot) are also reported in 2D TiO_2_ and WS_2_ [159, 160]. These heterojunctions undoubtedly demonstrate efficient charge separation, larger available surface area and enhance visible light absorbability, hence improving their photocatalytic performances compared to the individual components and non-2D nanostructures counterparts. Details of photocatalytic performances are found in Tables 1, 2, 3, 4, 5 and 6.

**Fig. 10:**
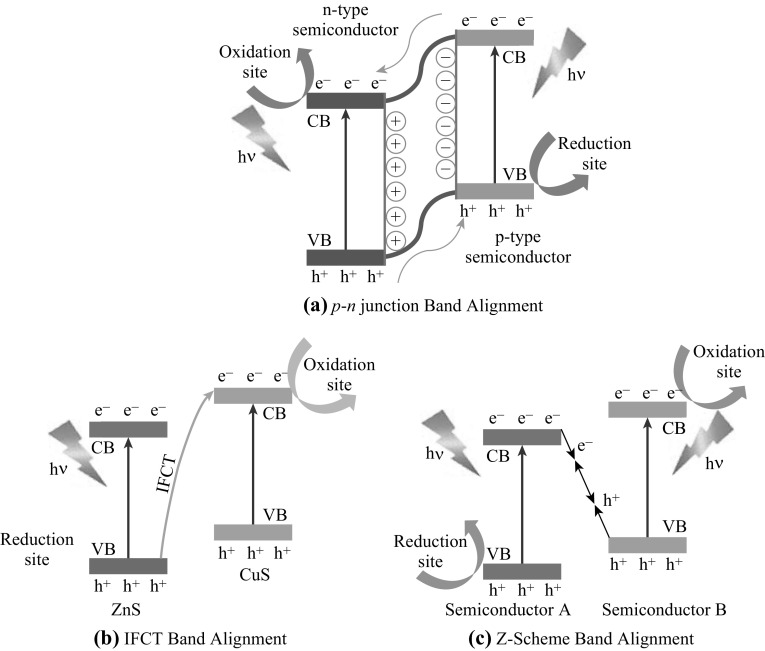
Schematics of heterojunctions of **a** *p*–*n* junction, **b** IFCT and **c** Z-scheme. Reproduced with permission from Ref. [164, 166, 216]

Apart from type II band alignment, the formation of *p*–*n* heterojunction is another effective approach to suppress the charge recombination [161]. In this approach, the contact between a *p*-type material with its *n*-type counterpart forms into a space charge layer and induces an internal electric field that can extend the probability of electron–hole separation (Fig. 10a). Meng et al. [162] fabricate a *p*–*n* heterojunction using *p*-2D MoS_2_ and *n*-2D reduced graphene oxide (rGO) to improve the photocatalytic activity of MoS_2_. Here, MoS_2_ not only acts as a catalytic center but also a photocenter for absorbing solar light to generate charge carriers. The *p*–*n* junction activated the photocatalytic H_2_ evolution performance of MoS_2_ although the production rate is still low (24.8 µmol (gh)^−1^) [162]. Being inspired by this work, Xing et al. [163] fabricate a *p*–*n* heterojunction based on *n*-CdS and *p*-CdTe nanocrystals to enhance the visible light absorbability and charge recombination suppression. As a result, *n*-CdS became an efficient electron collector. 2D Ti_0.91_O_2_ nanosheet is then introduced to the *p*–*n* heterojunction acting as an electron sink and providing HER catalytic sites upon the formation of a type II band alignment heterojunction with *n*-CdS. The corresponding production rate is 463 µmol (gh)^−1^, while no H_2_ evolution occurs for bare titanate nanosheets [163].

There are some special charge transfer mechanisms when specific materials are coupled with 2D TMO&Cs. Zhang et al. [164] develop a visible light-driven photocatalyst based on a 2D ZnS nanosheet/0D CuS nanocluster heterojunction. Upon visible light irradiation, the photogenerated electrons in the VB of ZnS are transferred directly to the CB of CuS clusters due to the interfacial charge transfer (IFCT) mechanism (Fig. 10b). Such a mechanism has been commonly seen in the TMC-Cu(II) complex system upon the hybridization between the discrete energy levels of Cu(II) molecule and the continuous ones of TMC. Subsequently, the transferred electrons cause the partial reduction in CuS to Cu_2_S and undergo effective reduction in H^+^ to produce H_2_ given that the potential of CuS/Cu_2_S is well above that of H^+^/H_2_O [164]. Meanwhile, the holes in the VB of ZnS are consumed by the sacrificial agents. As the result, this IFCT phenomenon retards the recombination of photoexciton pairs due to space separation. The optimal CuS loading is determined to be about 2 mol % and the corresponding H_2_ production rate is impressively ~ 4.2 mmol (gh)^−1^, which is eight times larger than that of 2 mol % Cu-doped ZnS nanoparticles [164].

The heterojunction composed of 2D ZnO and CdS nanoparticles also exhibits special charge transfer process, namely Z-scheme (Fig. 10c). The recombination of the photogenerated electrons from the CB of ZnO and holes from the VB of CdS occurs at the interface, resulting in the retention of the photogenerated electrons in CdS with a higher CB position and holes in ZnO with a lower VB position. It is proposed that the metallic polar surface of ZnO may be a key factor in response to initiate the Z-scheme transfer process [165]. Similar observation is seen in the 2D MoS_2_/0D Ag_3_PO_4_ heterojunction [166]. Conventionally, Ag_3_PO_4_ is an efficient photocatalyst but with weak chemical stability under prolonged light irradiation [68, 167]. When coupled with 2D MoS_2_, efficient separation of photogenerated electron–hole pairs in Ag_3_PO_4_ is observed as 2D MoS_2_ acts as an effective electron collector in the system. By comparing the band structures between MoS_2_ and Ag_3_PO_4_, a heterojunction with the type II band alignment is expected to form, but the electron transfer direction is actually opposite, i.e., from MoS_2_ to Ag_3_PO_4_. Zheng et al. propose that the charge transfer mechanism between MoS_2_ and Ag_3_PO_4_ is predominated by the Z-scheme mechanism as the electrons from the CB of Ag_3_PO_4_ recombine with holes from the VB of MoS_2_ using both the active species trapping and photoluminescence techniques [166]. With a small loading of MoS_2_, the heterojunction demonstrates an almost double enhancement on the RhB and ~ 50% enhancement on phenol photodegradation performances compared to those of bare Ag_3_PO_4_ [68, 166, 167]. Such a Z-scheme mechanism may be also applied to other Ag_3_PO_4_-based heterojunctions such as the recently reported 2D WO_3_/0D Ag_3_PO_4_ [168] and 2D Ti_0.91_O_2_/0D CdS heterojunctions [169].

#### Heterojunction with Conductive Materials

**Fig. 11:**
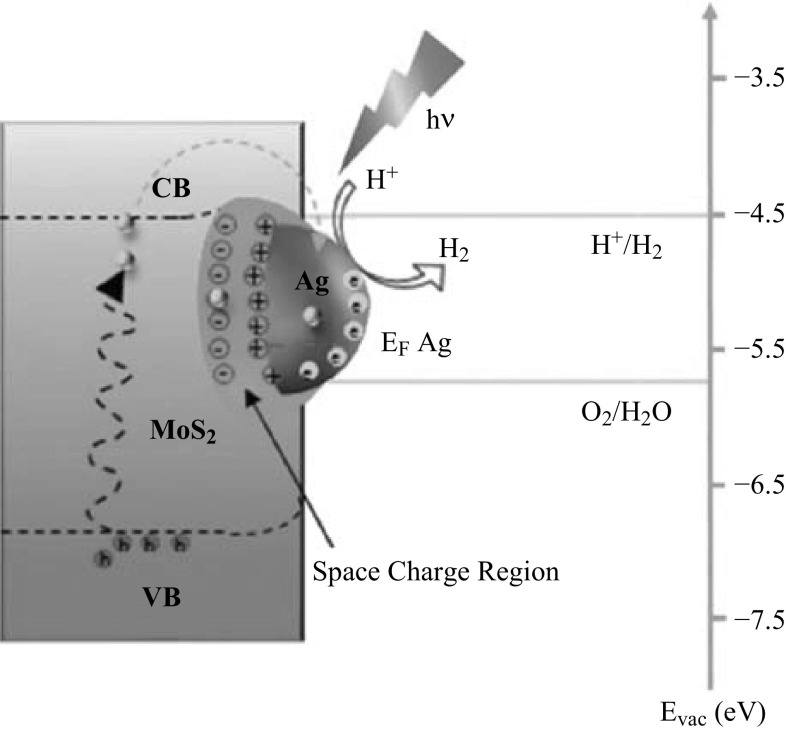
Illustration of heterojunction between a 2D MoS_2_ nanosheet and an Ag nanoparticle. Reproduced with permission of Ref. [175]

Similar to the heterojunctions formed with semiconductors, a rectified charge carrier transfer can occur in the Schottky barrier between a semiconductor and a metal, depending on their Fermi-level positions [170–172]. Upon light irradiation, electrons are generated in the CB of the semiconductor and lift its Fermi level to more negative values (Fig. 11) [173]. The resulted energetic difference at the semiconductor–metal interface drives the electrons from the CB of the semiconductor into the metal [173]. To incorporate into the heterojunctions with 2D TMO&Cs, the metals are either chemically co-synthesized with the semiconductor or synthesized individually first and then attached to the 2D TMO&C nanostructures for strong adhesion [174]. The metals are generally in the form of nanoparticle, and the weight ratios to the 2D TMO&Cs are optimized to avoid the surface overloading effect. In addition to the “electron sink” effect, some of the noble metal nanostructures exhibit localized surface plasmon resonance in the visible light spectrum, which can enhance the light harvesting efficiency of the heterojunction. In this case, the photogenerated electron–hole pairs are separated by the metal–semiconductor interface and the catalytic reactions hence take place on the surface of the plasmonic photocatalyst [175]. For instance, the incorporation of up to 1 wt% Ag nanoparticles into ZnO nanosheet results in an additional visible light absorption peak in the region between 400 and 500 nm, which is ascribed to the localized surface plasmon resonance generated by Ag nanoparticles. A MO degradation rate of 389.5 μmol (gh)^−1^ is demonstrated with ~ 50% enhancement compared to the ZnO nanoparticle counterpart [176]. Similar observations can be also seen 0.5 wt% Au–Pd/2D TiO_2_ nanosheets, 20 wt% Ag/2D MoS_2_ nanosheet as well as 1 wt% Cr/2D MoS_2_ nanosheet [175, 177, 178]. It should be noted that some noble metals such as Pt are good catalyst by themselves [179]. Careful investigation should be carried out when revealing the mechanism for enhanced photocatalytic performances from the noble metal–semiconductor heterojunctions.

Conductive carbon-based low-dimensional nanomaterials are also excellent alternatives for metals to form heterojunctions with 2D TMO&Cs [180]. Due to their high conductivity and electron mobility as well as large surface area, these materials exhibit even better charge separation efficiencies compared to those of metals in many cases. So far 0D carbon dots, 1D carbon nanofibers and 2D graphene nanosheet have been investigated for possible candidates in 2D TMOs (e.g., TiO_2_, Ti_0.91_O_2_ and Fe_2_O_3_) and TMCs (e.g., MoS_2_, WS_2_ MoSe_2_ and CoS) [98, 137, 160, 169, 175, 181–184]. Detailed photocatalytic performances of these heterojunctions are summarized in Tables 1, 2, 3, 4, 5 and 6. As mentioned in the previous section, due to the nature of heterojunction interface, the heterojunctions of graphene with 2D TMO&Cs can possess a relatively stronger electronic and physical coupling effect, resulting in remarkable enhancement in the electron transfer process across the heterojunction and yielding superior photocatalytic activity [69]. Indeed, graphene itself is an excellent charge transfer medium which slows the recombination of photoexciton pairs, thus increasing charge transfer rate of electrons and surface adsorbed amount of chemical molecules through the π–π interaction [185, 186]. Heterojunctioning graphene in 2D TMO&Cs is generally realized by hydrothermal/solvothermal and microwave irradiation synthesis approaches [136, 187]. During the synthesis, graphene oxide and transition metal salts are the initial materials, and then, both of them are reduced to graphene nanosheet and 2D TMO&Cs, respectively. Compared to 0D metal nanoparticle-based heterojunctions, the lateral dimension of graphene nanosheets is much larger than those of 2D TMO&Cs, leading to a large interfacial contact and hence improved electron transfer across heterojunction. In addition, the loading of graphene can be as large as 30–40 wt%, ensuring much better photogenerated charge separation efficiency [98, 181, 182, 185–187].

Recently, the utilization of metallic 2D TMCs in the heterojunction has attracted great interest. 2D MX_2_ (e.g., MoS_2_ and WS_2_) with metallic 1T crystal structures is typical representatives [55, 188]. As mentioned in the previous section, 1T 2D MX_2_ has a very high conductivity which exhibits improved charge transfer kinetics. More importantly, it provides additional active sites for H_2_ production on its basal plane, making it an efficient HER catalyst. When coupling with semiconductor as a co-catalyst, the electrons generated on the semiconductor can directly migrate to the basal sites for catalytic reactions, unlike the case for 2H MX_2_. This greatly shortens the diffuse length of electrons and hence reduces the chance of charge recombination [188]. Taking 1T MoS_2_, for example, the hydrogen production rate of 1T MoS_2_/TiO_2_ reaches 2 mmol (gh)^−1^, which is 5 and 8 times higher than those of bare TiO_2_ (400 µmol (gh)^−1^) and 2H MoS_2_/TiO_2_ (250 µmol (gh)^−1^), respectively [188].

#### Synthesis Techniques of 2D TMO&C-Based Heterojunction Photocatalysts

Physical mixture through grounding is the most straightforward approach to realize 2D TMO&C-based heterojunctions. However, the poor interfacial contact between materials is the major concern. Other physical approaches such as magnetic stirring and ultrasonication can improve the quality of the contacts [189–191]. Binding chemicals such as poly(ethyleneimine) (PEI) are also used for inducing higher degrees of adhesion in the heterojunctions during the hydrothermal/solvothermal and chemical bath synthesis [98, 162, 164, 176, 177, 181, 188]. Interestingly, there are special synthesis approaches for particular heterojunctions. Examples include ion exchange method for 2D ZnS/Ag_2_S by replacing Zn^2+^ with Ag^+^ [192], layer-by-layer self-assembly technique for 2D Ti_0.91_O_2_/CdS using poly(methyl methacrylate) (PMMA) spheres as the template [169, 187] and calcination of TMCs in an oxygen-rich environment for creating 2D WS_2_/WO_3_ and MoS_2_/MoO_3_ contacts [112, 155]. Microwave irradiation synthesis is reported to be highly effective in forming heterojunctions with graphene and carbon dots [160, 187], while high-power UV irradiation can transform adsorbed noble metal ions into nanoparticles on the surface of 2D TMOs during the semiconductor–metal heterojunction formation [174].

## Summary and Outlooks

The application of both layered and non-layered 2D TMO&Cs in photocatalysts has received rapid momentum in recent years. The properties of 2D TMO&Cs have shown distinct advantages for photocatalytic HER, pollutant degradation, carbon reduction and microbial disinfection process. Through a significant number of demonstrations, the larger surface area of 2D nanostructures and their 3D derivative hierarchical structures offer more reaction sites for photocatalytic reactions and facilitate the charge migration for improving photocatalytic reaction kinetics. The unique tunable band structures in 2D TMO&Cs lead to more thermodynamically favorable redox reactions during the photocatalytic processes. Furthermore, the predominant exposed crystal facets provided by the 2D geometrical configuration also greatly improve the photocatalytic performance.

The investigation of 2D TMO&C-based photocatalysts is still in its early stage compared with the relatively mature 1D and nanoparticle-based photocatalytic systems. Many opportunities are just emerging, and significant advances are expected in the near future. The key for accelerating the research field is to produce 2D TMO&Cs especially with the non-layered crystal systems in a facile and high-yield manner. The further optimization of deposition parameters for vapor-phase synthesis, such as the amount of source material and vaporization energy/temperature, is needed to conduct for maximizing the production yield that is suitable for efficient photocatalytic reactions. Through possible implementation of novel templates and grow directing agents, liquid-phase synthesis techniques can also offer great opportunities for large scale production of 2D TMO&Cs with strong morphological controllability, which will be of great importance for developing efficient 2D TMO&C-based photocatalysts met with the industry standard.

The improvement strategies of photocatalytic performances are also critically discussed in this paper, particularly focusing on the approaches of elemental doping, surface functionalization and heterojunctions with both semiconducting and conductive materials. Compared to other 2D/low-dimensional heterojunctions, the 2D/2D system is suggested to exhibit greater stability and better coupling heterointerfaces, which facilitates the photoinduced charge transfer and separation. However, the related synergetic effects are currently less studied and should be further explored for designing new 2D/2D heterojunctions with extraordinary photocatalytic properties. In addition, the tuning of stoichiometry in 2D TMO&Cs can greatly influence their electronic band structures, resulting in narrowing bandgap energies and possibly producing surface plasmon resonance in the visible light region when their free charge carrier concentrations are large enough. The implementation of the sub-stoichiometric phase of 2D TMO&Cs in photocatalytic applications can possibly offer various benefits including the improvement of the light harvesting and photoexcited charge carrier transfer.

All in all, possibilities of new insights in 2D TMO&C-based photocatalysts are plenty and require significant resources and attention to discover new phenomena that harness the unique strength provided by 2D materials.
